# Briefest Time to Perform a Series of Preoperative Nerve Blocks in Multiple Patients: A Simulation Study

**DOI:** 10.7759/cureus.16251

**Published:** 2021-07-08

**Authors:** Richard H Epstein, Franklin Dexter, Jeffrey J Mojica, Eric S Schwenk

**Affiliations:** 1 Anesthesiology, University of Miami Miller School of Medicine, Miami, USA; 2 Anesthesia, University of Iowa, Iowa City, USA; 3 Anesthesiology, Thomas Jefferson University, Philadelphia, USA

**Keywords:** computer simulation, monte carlo method, spinal anesthesia, conduction anestehsia, anesthesiologists, preoperative period, healthcare quality assurance

## Abstract

Introduction

To mitigate first-case delays in operating rooms, sufficient additional time must be allotted when anesthesiologists perform preoperative nerve blocks in multiple patients who are scheduled as the initial cases of the day. We used spinal anesthetics performed in dedicated block rooms located just outside the operating room suite to estimate the briefest times needed to complete a series of spinal, epidural, peripheral, or other regional nerve blocks. We followed this approach because even though the studied hospital had a busy regional anesthesia service, sample sizes were insufficient and electronic data were not available to directly study the time to perform the many other nerve blocks they perform.

Methods

We studied a historical cohort of 8,462 adult patients undergoing spinal anesthesia between 2005 and 2017. Preoperative evaluation, consent, and holding area tasks were completed before entering the block room; the times to complete these tasks were not available for study. Upon block room entry, the electronic anesthetic record was started, a timeout conducted with patient participation, vital signs taken, and the spinal performed. The interval from entry until intrathecal injection was the spinal block time. Because fits of these times to probability distributions previously used for anesthesia times were poor (p < 0.001), percentiles of times to perform one or more spinal anesthetics were calculated using Monte-Carlo simulation (100,000 samples with replacement) from the empirical distributions.

Results

The mean spinal block time was 8.8 minutes. The 90% upper prediction limit for one block was 14 minutes, with progressively decreasing times for each subsequent block for a 90% chance of finishing on time. For example, for three first-case regional or neuraxial blocks performed outside the operating room by one anesthesiologist, the first patient needs to arrive at least 38 minutes earlier than non-block patients to mitigate operating room start delays.

Conclusions

These minimum time estimates can help nursing leadership ensure that sufficient time will be available after patients are ready for anesthesia to avoid first-case delays when preoperative regional anesthesia is performed outside the operating room. Given that inadequate sample size and documentation issues likely exist universally for the various non-neuraxial preoperative nerve blocks, we recommend that hospitals use our estimates as a minimum starting point rather than try to calculate times using their own data. Then, as a systems-based metric to assess all steps in the process, track the percentages of days for which all blocks were completed in sufficient time to avoid a first-case delay for those patients. Adjustments to the arrival times would then be implemented, if needed, to meet hospital objectives for on-time starts.

## Introduction

Increasingly, preoperative nerve blocks are being placed either as the primary anesthetic e.g., spinal anesthetics for lower extremity joint replacement [1] or regional blocks for vascular access surgery [2] or postoperative analgesia such as interscalene blocks for shoulder surgery [3]. When such blocks are requested for the multiple patients scheduled as the first cases of the operating room (OR) day, sufficient time beyond that required for routine preoperative processes completed before entering the ORs should be planned by nursing administrators to mitigate first-case delays. We are unaware of previous studies that guided forecasting the briefest amount of additional time that should be allotted beyond that planned for surgical patients not receiving such blocks.

Addressing such projections analytically is impractical for many departments. There are insufficient sample sizes for individual or sequences of the various blocks, difficulty in obtaining accurate data from the electronic health record, and a lack of knowledge as to what statistical distribution to use for the block times. There are also statistical challenges in making the projections. Despite the presence of a busy regional anesthesia service at the study hospital, sample sizes for non-neuraxial preoperative blocks were grossly inadequate for analysis. Also, these blocks were usually documented on paper without sufficient timestamps to determine how long they took to perform. Because we had very large sample sizes for preoperative neuraxial nerve blocks with accurate electronic documentation, we, therefore, estimated the briefest amount of time required to perform a series of blocks using simulation.

The objective of our study was to model a series of nerve blocks for calculation of the 90% upper prediction limit of the time to complete the series by one anesthesiologist or by two anesthesiologists working in parallel. To perform the simulations, we took advantage of a large cohort of patients at an academic hospital who received spinal anesthetics in dedicated block rooms before entering an OR and in whom documentation times were captured in the electronic medical record. We use reliably obtained spinal block times from individual nerve blocks (i.e., from block room entry to intrathecal injection of the local anesthetic) to estimate, using simulation, the briefest amount of time that needs to be set aside for the regional anesthesia team to perform a series of nerve blocks for first cases of the day. The briefest time is what is necessary for communication with nursing teams, in that having the patients ready later than required predestines that the anesthesiologists will not reliably have blocks performed without having first-case delays. Nursing administrators would use this value when considering the arrival time to be communicated to patients anticipated to receive a preoperative nerve block before entering the OR. This value could also be used for improved communication with perioperative nursing as to the importance of prioritizing the preparation of these patients to help avoid delays [4]. Because most peripheral nerve blocks take longer to perform than a spinal [5-7], we used the data and simulation to estimate the briefest time that should be anticipated when peripheral nerve blocks (e.g., ultrasound-guided brachial plexus blockade) are to be performed in a series of patents.

## Materials and methods

Data source

The Thomas Jefferson University Institutional Review Board approved this retrospective, database study as exempt (i.e., not requiring patient consent) on March 24, 2021 (Control #21E.246). We studied a historical cohort of 9,091 spinal anesthetics performed for lower extremity joint arthroplasty in dedicated block rooms located within the surgical suite between October 18, 2005, and March 17, 2017, using deidentified records from the hospital’s anesthesia information management system database (Innovian, Dräger Medical, Telford, PA). This system was used to create the anesthesia record, including care delivered in the block rooms. This date range corresponded to the entire period during which the system was in use. Data used included the arrival time in the block room, the time of injection of the intrathecal anesthetic, and the category of the primary anesthesia provider for the case. Data regarding whether the patient was seen preoperatively were linked to the vendor’s preoperative evaluation system, written in LiquidOffice (OpenText, Waterloo, Canada), based on the unique identifier of the surgical case. Data from Innovian have been used for approximately 50 previously published studies from the hospital studied and demonstrated to be of high quality [8]. The primary author (RHE) had access to the database and wrote the code to extract and analyze the data. We followed the Equator Network guideline “The reporting conducted using observational routinely-collected health data (RECORD) statement” in conducting this study.

Study population

The vast majority (96.1%) of the patients had been fully evaluated in the hospital’s preadmission testing center before the date of surgery, including a physical examination and history, relevant consultations, and laboratory testing. All charts and results were reviewed and cleared by one of the anesthesiologists who worked in the center, and additional follow-up arranged, as indicated.

Among the 9,091 patients studied, all of whom were adults, 629 cases were excluded due to missing or internally inconsistent data (6.92%). Reasons for exclusion included the absence of documentation of the time of intrathecal administration in the drug dose field (n =235), the time of administration was before entering (n = 156) or after leaving (n = 174) the block room or after entering the OR (n = 117), or the time leaving the block room was after the time entering the OR (n = 73); 190 patients had more than one exclusion criteria. After exclusion, 8,462 patients who received spinal anesthesia in a block room were available for study.

Among the 8,462 patients studied, there were 43 inpatients (0.51%) who were seen by a resident before surgery using the same electronic preoperative evaluation system as used in the preadmission testing center. Under the medical direction of an anesthesiologist, Certified Registered Nurse Anesthetists (CRNAs) performed 88.5% of the spinal anesthetics, with the remainder done by residents, student nurse anesthetists; no anesthesiologist assistants were involved. If there was difficulty performing the block, the anesthesiologist did it. The anesthesiologists and CRNAs involved in these arthroplasty cases were usually members of the orthopedic surgery anesthesia team and highly proficient in neuraxial anesthesia.

On the day of surgery, patients were registered and prepared for surgery in a preadmission unit, then transported to the holding area, where additional nursing and anesthesia processes were completed, including chart review, physical examination, and obtaining the anesthesia consent (if not secured in the patient testing center), and intravenous catheter placement. Upon arrival in the block room, the electronic anesthesia record was started, which commenced the automatic recording of vital signs. A timeout was performed before each neuraxial block with active participation by the patient. The patient was then positioned, usually in the sitting position, but occasionally in the lateral decubitus position, and the spinal was performed. A registered nurse trained in assisting during neuraxial anesthesia helped with the positioning. The interval from patient entry into the block room to the time of intrathecal injection of the local anesthetic was defined as the spinal block time.

Statistical analysis

We attempted to model the spinal block times to various distributions that have been used previously to model anesthesia times, including the normal distribution [9], two-parameter log-normal [10,11], gamma distribution [12], and Weibull distribution [13]; we used Systat version 12 (Systat Software, San Jose, CA). If a known distribution showed a good fit to the data, then we would have calculated the prediction intervals using the parameters of the theoretical distribution [11]. However, all fits were poor, from inspection of the graphs, and all the Kolmogorov-Smirnov tests’ had p < 0.001, even though those tests were fits to models with known parameters (ie, overestimates of p-values to distributions with estimated parameters).

Therefore, to compute the percentiles of the times to perform one or more spinal blocks, we relied upon the empirical distribution of the spinal block times. These percentiles were calculated using Monte-Carlo simulation in Office 365 Excel (Microsoft, Redmond, WA) with 100,000 samples taken with replacement. For example, to calculate the percentiles of the times to perform three spinal blocks, three uniform random numbers were generated using the Mersenne Twister algorithm. Each uniform variate was used to select (with replacement) from among the 8,462 observed spinal block times, sorted in ascending sequence. The sum was then taken of the three random observations for each of the 100,000 samples. Because the sample sizes are extremely large, the 90th percentiles are 90% upper prediction limits for future observations. The 90% upper prediction limits are the endpoint used in OR management science for reducing tardiness of starts of OR cases [11,14].

When there are many preoperative blocks to perform in first-case patients (e.g., more than three or four), two anesthesiologists might be assigned to work in parallel. To assure a 90% chance that both anesthesiologists complete their series of blocks within a forecasted time, we calculated the 95% upper prediction limit of the time for one anesthesiologist to complete that number of blocks, where 95% = 100 × (1-0.10/2). The 95% upper prediction limit is analogous to 90% with Bonferroni adjustment for two comparisons.

Simulation results with 100,000 simulations were compared with results from the first 90,000 samples. The maximum absolute differences for the 95% upper prediction limits were all less than four seconds (ie, unimportantly small errors). Thus, a sample size of 100,000 simulations was sufficient.

Studying spinal blocks was especially useful because this procedure is quick to perform, and the times to complete those blocks were recorded in a room designed and used exclusively for nerve blocks and with dedicated personnel. More complicated blocks would be expected to take longer. To explore this, we had another group with >1,000 combined spinal-epidural (CSE) performed in the same block rooms and with the same anesthesia personnel (ie, a valid comparison). Between October 18, 2005, and June 30, 2011, a CSE was often placed for knee arthroplasty; this technique was largely abandoned in July 2011. After this date, peripheral nerve blocks, mostly placed postoperatively in the postanesthesia care unit, became the preferred technique for postoperative analgesia in knee arthroplasty patients. The CSE patients underwent the same preoperative processes as those receiving spinal anesthetics for lower extremity joint arthroplasty. The 8,462 times to place spinal block were compared to the times for the 2,916 CSE blocks using the Wilcoxon-Mann-Whitney signed ranks test. This test was done to confirm the expected finding that blocks more complicated than a spinal take longer to perform [5-7].

## Results

Among the 8,462 spinal anesthetics studied, the mean block time was 8.8 minutes (standard error (SE) = 0.06 minutes) from block room entry to intrathecal injection of the local anesthetic. Because the time to perform a given spinal block is independent of the time to perform another block, the mean times of a series of spinal blocks are additive [15]. That is, the average time to complete five simulated blocks was 5×8.8 = 44 minutes. However, what is relevant for planning purposes is to provide a sufficient interval so there is a 90% chance of completing the series of blocks within that interval [11,14]. For example, if the anesthesiologist has four blocks to perform, 49 minutes would be planned (Table 1). If two anesthesiologists were working in parallel to perform six blocks (ie, each performing three blocks), then 43 minutes would be planned (Table 1).

**Table 1:** Times (minutes) to perform various numbers of sequential spinal anesthetics UPL: Upper Prediction Limit

# Spinals	Median	Mean	80% UPL	90% UPL of time for one anesthesiologist	90% UPL of time for two anesthesiologists each performing the # of spinals in parallel
1	8	9	11	14	18
2	16	18	22	27	32
3	25	26	32	38	43
4	33	35	42	49	55
5	42	44	52	59	66
6	51	53	62	70	77
7	60	62	72	80	87
8	68	71	81	90	98
9	77	79	91	100	108

**Figure 1:**
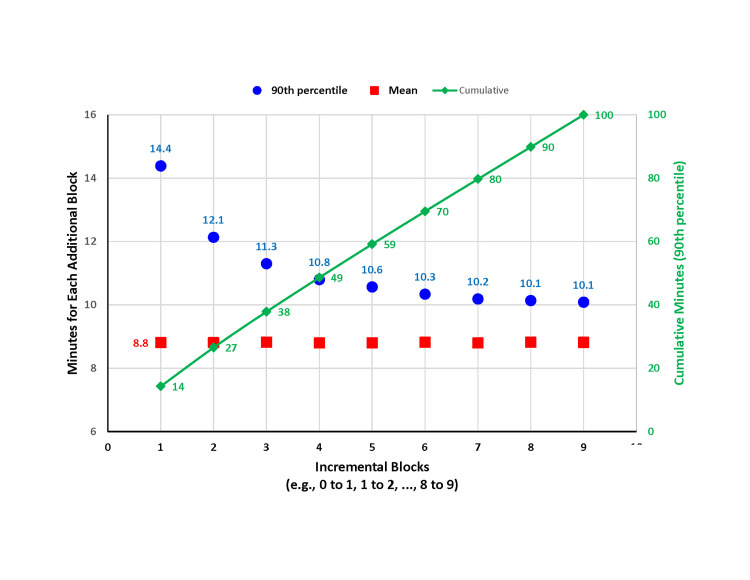
Times to perform a series of spinal anesthetics. The squares indicate the average time to perform a spinal anesthetic; this was the same for each individual block. The circles indicate the 90th percentile of the incremental time for each of the blocks. For example, the 90th percentile upper prediction limit (UPL) of the time to perform one spinal was 14.4 min, and the 90th percentile UPL to perform the second spinal was 12.1 min. The 90th percentile of the cumulative time (diamonds) to perform the indicated number of spinals matches column 5 of Table 1; for example, a total of 38 min for one anesthesiologist to perform a spinal in three consecutive patients. The squares and circles correspond to the differences between successive rows in Table 1 for the mean and the 90% UPL of the time for one anesthesiologist, respectively.

The 90% upper prediction limit of the time to complete one block was 14.4 minutes, but the incremental time for each additional block, while maintaining a 90% chance of completing the additional block within the cumulative amount of specified time, was progressively less (Figure 1). For example, if the anesthesiologist has three peripheral nerve blocks to perform for first-cases, he or she would need at least 38 minutes to have a 90% chance of completing the series, assuming that there were no delays between the time ending with one patient and starting with the next patient (Figure 1). Thus, the first patient to be blocked would need to arrive at least 38 minutes earlier than typical for first-case patients not undergoing a preoperative regional block outside the OR. Note that 38 minutes is longer than 26.4 minutes = 3×8.8 minutes (mean for one block) but less than 42 minutes = 3×14 minutes (90% upper prediction limit for a single block, Table 1).

In practice, because most other blocks typically performed by regional anesthesia services are more complicated, and thus take longer than a spinal, even more, lead time than the minimum estimated from the simulations would need to be provided. To confirm the expectation that more complicated blocks than spinals do take longer (see Methods), we evaluated the 2,916 patients who underwent CSE in the dedicated block rooms, typically for knee arthroplasty. These blocks took more time to perform than the spinals (12.8 minutes versus 8.8 minutes, SE = 0.12 min, p < 0.0001). One can see in Table 2 that all statistics for CSEs were longer than for spinals (Table 1). For example, for one anesthesiologist to perform three CSE blocks, 53 minutes would be needed to ensure at least a 90% chance of completing the series within that interval, compared to 38 minutes for three spinals.

**Table 2:** Times (minutes) to perform various numbers of sequential combined spinal-epidural anesthetics CSE: Combined Spinal-epidural; UPL: Upper Prediction Limit

# CSE	Median	Mean	80% UPL	90% UPL of time for one anesthesiologist	90% UPL of time for two anesthesiologists each performing the # of CSE in parallel
1	12	13	17	21	25
2	24	26	32	38	43
3	37	39	47	53	59
4	50	51	61	68	75
5	63	64	75	83	91
6	75	77	89	98	106
7	88	90	103	113	121
8	101	103	117	127	136
9	114	116	131	141	151

## Discussion

When performing a series of preoperative nerve blocks outside the OR, avoiding delays in first-case starts requires nursing administration intervention to ensure that such patients arrive at the hospital in sufficient time to complete all preoperative tasks with additional time allotted for the nerve block [16]. Our study provides an estimate of the briefest amount of time that needs to be provided to ensure sufficient time to perform all the scheduled blocks on 90% of days. The briefest time is the value needed because patients being ready any later will be insufficient for reliable on-time starts. Patients who will undergo a preoperative nerve block should be flagged so that administrative tasks and nursing care can be prioritized. We emphasize that actual times for a series of preoperative nerve blocks will almost certainly exceed our estimates. For example, in our study, separate consent for the spinal anesthetic did not need to be obtained because that was the primary anesthetic and included in the informed consent obtained before the patient entered the block room. For many other nerve blocks, the regional anesthesiologist would need to obtain separate consent, adding time to the process. We also do not include the time necessary to prepare routine patients on the day of surgery because those would be incorporated into existing processes related to when such patients are told to arrive. The reason that our calculated minimum times are useful is because, for multiple blocks they are substantial, requiring communication with nursing departments [17].

Given that peripheral nerve blocks usually take longer to perform than spinals, even more lead time likely will be required than provided by our simulations. For example, Chelly et al. studied various peripheral blocks in 549 patients and determined that the mean time from the arrival of the regional anesthesia provider in the patient’s room until the patient was repositioned after the block was 21 minutes [7]. Given that our patients receiving spinal anesthesia were immediately placed in the supine position after intrathecal injection of the local anesthetic, their measurement points seem comparable to those in the current study, where a shorter mean time of nine minutes was observed. However, we lack insight into how their 21-minute mean time would translate into a 90% upper prediction limit because the block times we studied could not be modeled by any of the standard probability distributions used for such predictions. A review of the relevant literature, comprising scores of publications, identified no studies that reported using one of the standard distributions, as compared with rank-based analyses (e.g., Wilcoxon-Mann-Whitney tests). Thus, our findings of lack of fit to a probability distribution are typical, not unusual. Consequently, we modeled the times for a series of blocks using the empirical distribution of spinal block times from a very large sample size.

We recommend that future scientific studies of nerve blocks accurately capture timestamps related to the continuous time that the regional anesthesia team directly cares for the patient to refine the lead times needed. Nevertheless, the important lesson for clinicians considering the novelty of our work is that it is not practical to use individual hospital data to estimate the times to complete the series of blocks without thousands of cases of each block type. The percentage of ORs with on-time starts cannot be modeled realistically to choose patient arrival times because of the absence of matching probability distributions. However, what can be done for quality monitoring is to record the percentages of days for which all blocks were completed in sufficient time to avoid a first-case delay for those cases. Such monitoring would be a systems-based approach to assess all steps in the process for these patients, analogous to the valid monitoring of the percentages of days with no delays in starting urgent cases or days with no delays in phase I postanesthesia care unit admissions [18-21]. Adjustments to the arrival times would then be implemented, if needed, to meet hospital objectives for first-case on-time starts.

Our work is useful because hospitals have strong biases towards the importance of first-case on-time starts, and these biases change decision-making [22-24]. If sufficient time is not allotted for the performance of a regional anesthetic, one should anticipate that there often will be avoidable first-case delays in these cases. However, it is commonplace for personnel arranging for patient-ready times in operating rooms to plan too little preparatory time, which is the value to our work [17].

We address factors attributable to suboptimal nursing administrative practices. This is important for two reasons. First, regarding individual patients being ready, the regional anesthesia service should not be held accountable for first-case delays resulting from a failure to ensure that patients are ready for the regional anesthesia team sufficiently early to enter the OR at the scheduled time. Second, Lapierre et al. showed a cascading organizational effect on improved first-case starts from nursing having patients available on-time [4]. Following this improvement, the average number of minutes of tardiness per case attributable to the anesthesiologists decreased, followed by a decrease attributable to the surgeons [4].

Logistical issues

Implementing the process we describe depends on determining, in advance, which patients with first-case starts are likely to receive a preoperative nerve block. This could be done if the block were included with the scheduled procedures when the surgeon schedules the case. Alternatively, if the regional anesthesia service were to identify the patients who are block candidates, they would need to do this after the OR schedule is finalized because there are many changes to the final OR schedule up to the afternoon before surgery [25-27]. This information would then be used by the individuals calling patients with their required arrival time.

Optimizing workflow for the performance of preoperative blocks might also involve changes in staff scheduling in the holding area, prioritizing patient arrivals from registration areas, and ensuring that surgeons and anesthesiologists will be present to allow the regional anesthesia team sufficient time to perform their blocks. If the workflow incurs delays upstream from being able to perform the regional block (e.g., there is no surgical consent, the chart is incomplete, insurance authorizations are missing, physicians arrive late), the additional time planned may be wasted if the regional team has to wait for such issues to be addressed.

Limitations and strengths

A limitation of our study is that it was from a single hospital where the entire process of providing care for the high-volume joint arthroplasty surgeons was highly optimized to avoid delays [28,29]. Patients were nearly always well-prepared before the day of surgery, with their charts in order, all labs and studies resulted, consents on the chart, and any medical issues addressed before the day of surgery. Thus, there were minimal delays related to administrative issues and rarely patient medical problems related to inadequate preoperative evaluation to address on the day of surgery. The joint arthroplasty rooms typically started an hour before the main operating rooms and the corresponding patients were brought in early. The anesthesia providers performing the spinals comprised a small group of anesthesia attendings and nurse anesthetists adept in neuraxial techniques and worked regularly with the orthopedic surgeons. A trained registered nurse was provided to assist with the blocks, and three dedicated, fully stocked block rooms were available. Because the joint arthroplasty service was extremely busy and a major source of hospital revenue, there was a great deal of administrative interest in ensuring that the process flowed smoothly. Surgeon requests to modify personnel or the workflow to reduce their perceived sources of delays were frequently accommodated.

A consequence of these deliberate limitations is that times at most other hospitals will be longer than the briefest additional times we estimated to allot for patients receiving preoperative blocks. This will especially be true if local processes during the performance of regional blocks are more extensive than at the hospital studied, if supplies are not readily at hand in the block rooms and need to be retrieved, if personnel to assist in positioning are not dedicated to that role, or if there are other factors preventing the seamless transition from one patient to the next. However, these additional sources of delay further highlight the importance of bringing patients who will receive a preoperative nerve block into the hospital earlier than typical for patients not receiving such procedures.

A strength of the study is that the need to provide additional lead time is also relevant to situations where the anesthesiologist supervising the case is placing nerve blocks outside the OR in several first-case patients (e.g., covering multiple joint arthroplasty rooms). That anesthesiologist still needs to perform all the routine preoperative tasks and will be unable to do so while physically present for the regional or neuraxial block.

## Conclusions

Lead times above what are provided routinely must be planned by nursing leadership to avoid first-case delays when preoperative regional anesthesia is performed outside the OR, especially when multiple patients are involved. Our estimates for the briefest time required for a specified series of regional blocks performed by either one or two anesthesiologists provide guidance on how far arrival times need to be moved up. Prioritization of preoperative care of these patients is also necessary to avoid administrative delays that would impede the continuous workflow of the regional anesthesia team. As a systems-based metric to assess all steps in the process, we recommend tracking the percentages of days for which all blocks were completed in sufficient time to avoid a first-case delay for those patients. Adjustments to the arrival times would then be implemented, if needed, to meet hospital objectives for on-time starts.
